# Is antimicrobial resistance evolution accelerating?

**DOI:** 10.1371/journal.ppat.1008905

**Published:** 2020-10-22

**Authors:** Christopher Witzany, Sebastian Bonhoeffer, Jens Rolff

**Affiliations:** 1 Freie Universität Berlin, Institut für Biologie, Evolutionary Biology, Berlin, Germany; 2 Institute of Integrative Biology, ETH Zürich, Zürich, Switzerland; 3 Berlin-Brandenburg Institute of Advanced Biodiversity Research (BBIB), Berlin, Germany; University of Utah, UNITED STATES

Globally, antimicrobials are a main pillar of medical, veterinary, and agriculture interventions [1,2]. In all cases, resistance of microbes against antimicrobials is prevalent. The problem is exacerbated by the drying up of the antibiotic pipeline, as economic incentives to develop new drugs are very limited. In antifungals, the range of available compounds is also low with only 4 main classes of drugs available to treat fungal infections in humans and 6 main classes used in agriculture, with 1 class, the azoles, used in both [1].

The problem of drug resistance evolution has been observed early on in the antibiotic era [3,4]. Ultimately, however, the introduction of each antimicrobial resulted in resistance evolution in target and nontarget microbes. In realization of this problem, some antibiotics such as daptomycin were even developed with avoiding resistance evolution in mind, yet it took only 2 years from the introduction of daptomycin until resistance was recorded [4]. But how fast is resistance evolving?

Here, we want to discuss how fast resistance emerges after the introduction of antimicrobials. We base this on widely cited data in the literature for antibiotics ([4–7]; see also Fig 1A, based on [8]) and compared this to data on antifungal resistance [9,10]. Replotting the antibiotic data (Fig 1B), by displaying the time from introduction to resistance emergence over the year of introduction, suggests that the evolution of antibiotic resistance is accelerating over time. The same trend can be observed for antifungals (Fig 1C and 1D). In the following, we focus on (1) the quality of the underlying data and (2) possible explanations for this pattern of accelerating resistance.

**Fig 1:**
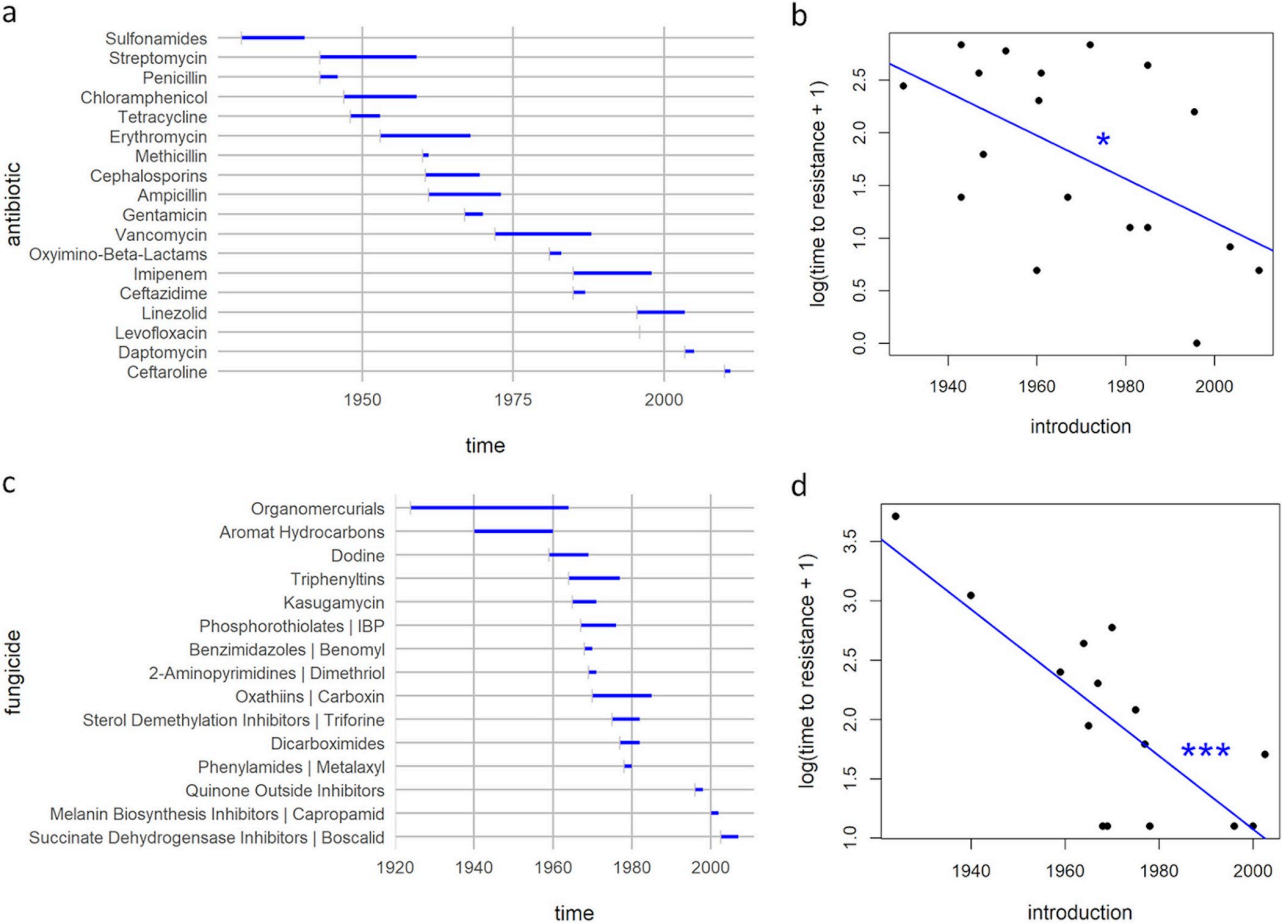
Timeline of antibiotic (a) and antifungal (c) introduction and detection of surmised evolved resistance depicted by the ends of the bars. In antibiotics (b) and antifungals (d), the time to resistance decreases with the year of introduction: for newer antimicrobials, resistance is described earlier after introduction (linear regression for antibiotics (b) F_1,16_ = 6.47, *p* < 0.05*, R^2^ = 0.29 and antifungals (d) F_1,13_ = 19.57, *p* < 0.001***, R^2^ = 0.60).

We first want to ask how reliable these commonly presented data on resistance emergence are. For this, we tried to trace the original papers from which the aggregated data for antibiotics (using [7] as a starting point) and antifungals [9,10] were obtained. To our great surprise, finding the original data for the antibiotics was very difficult, and many of the original sources could not be verified (see S1 Fig and S1 Appendix for more details). While the pattern of accelerating resistance in antibiotics might be true, it certainly cannot be supported given the data currently available. By contrast, for the antifungal data, it is possible to identify the original publications in most cases (see S2 Fig, S2 Appendix, and S1 Table). Replotting the suspected relation with the data that can be traced shows that the pattern of accelerating resistance evolution still holds for antifungals (Fig 2). Given that the antifungal data with traceable sources show a clearly accelerating trend, this should certainly be investigated for antibiotics.

**Fig 2:**
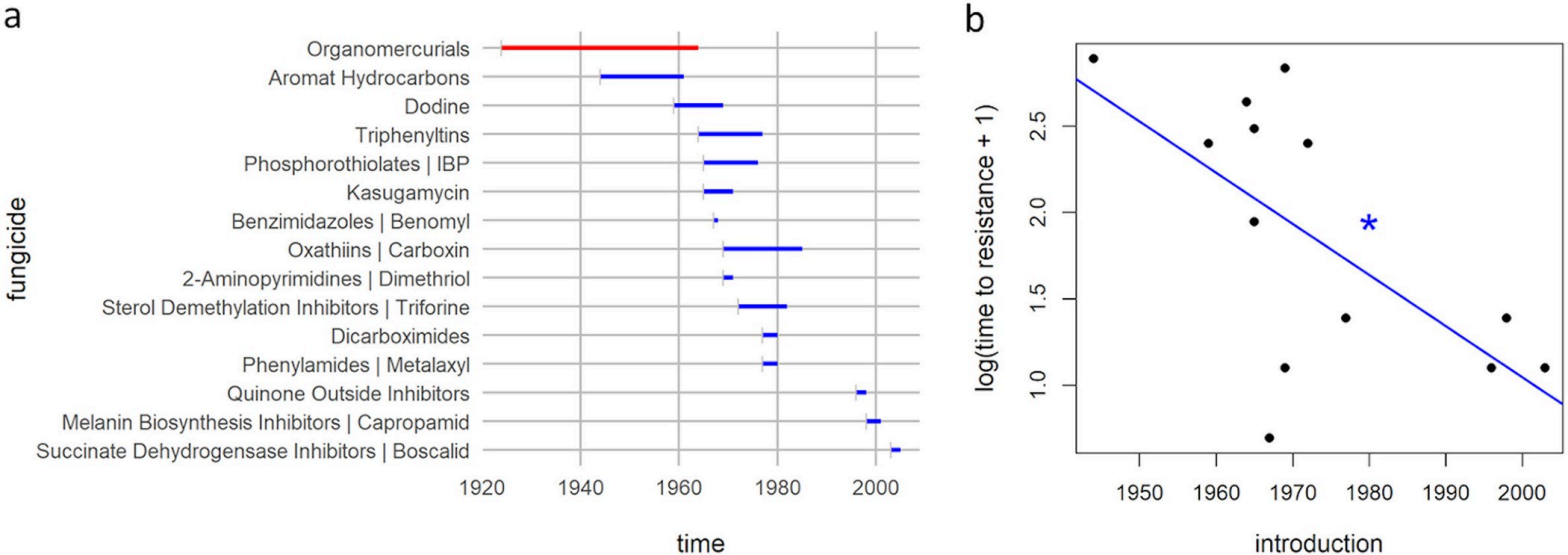
Timeline of antifungal (a) introduction and detection of evolved resistance for antifungals where the source could be traced (unambiguous and reliable sources of data points are shown in blue; untraceable data are shown in red and excluded from analysis) (b). The time to resistance decreases with the year of introduction (linear regression F_1,12_ = 8.10, *p* < 0.05*, R^2^ = 0.40).

If we take the data for both antibiotics and antifungals at face value, we see a clear trend of accelerating resistance evolution. We briefly want to propose 3—mutually not exclusive—testable hypotheses: (1) increase in usage; (2) increase in surveillance; and (3) evolutionary dynamics: cross-resistance, concurrent selection, and environmental enrichment of resistance genes.

## Increase in usage

Since the introduction of both antibiotics and antifungals, their use has steadily increased. This results in higher amounts of antibiotics, antifungals, and their residues in the environment, and this problem is exacerbated by the projected increase in global usage (e.g., [11]). That usage per se is correlated with prevalence of resistance is well established [12]. However, the hypothesis would predict more specifically that antibiotics and antifungals that have been introduced later are used more, because the pattern we observe is that resistance emerges faster for later introductions. Notably, some of the antibiotics concerned are reserve antibiotics, for which we expect that the usage is limited. Therefore, while testable, we think that this hypothesis may not be the most likely explanation for the timing of resistance emergence.

## Increase in surveillance

At the same time when the use of antibiotics and antifungals intensified, a parallel increase in surveilling infections and infection outcomes, and equally pest control measures, can arguably result in a higher probability of resistance detection. If this were true, it might well be sufficient to explain the pattern of accelerating resistance. In fact, if this was the sole explanation, it would be reassuring. It would mean that the older the data, the higher the probability that resistance was not detected when it evolved. In support of this notion, the number of publications on antibiotic resistance is increasing year to year [13].

## Evolutionary dynamics: Cross-resistance, concurrent selection, and environmental enrichment of resistance genes

Many newer antibiotics and antifungals are variations on older substances. Moreover, almost all of the antibiotics are derived from natural antibiotics. Therefore, resistance evolution might become faster, because growing reservoirs of resistance genes and resistant microorganisms in the environment can cause cross-resistance [14,15]. Also, some mutations conferring resistance reduce the fitness costs of genes providing resistance against other antibiotics [16]. Moreover, antibiotic resistance, despite often being costly, can persist in the environment in the absence of the selective agents [17,18]. This applies both to the persistence of resistant bacteria as well as the persistence of antibiotic resistance genes and can be caused by several mechanisms that mitigate the costs [17] or concurrent selection, for example, by heavy metals [14] or by pesticides [19]. Finally, as resistance mechanisms against different antibiotics might be under co-selection mediated by genetic linkage [20], the increase in resistance genes, resistant microbes, and antibiotic residues will facilitate faster resistance evolution. Many of the mechanisms mentioned for antibiotics and possibly others such as increase in mutagenesis or evolution of bet-hedging almost certainly apply to antifungals as well and can be seen as combining to reduce the available genomic resistance space. The risk of emergence of resistant nontarget species of agricultural fungicides is illustrated by potential cross-resistance of *Cryptococcus gattii* against agriculturally used benomyl and clinically used azoles [21].

We think that each of these hypotheses, especially (2) and (3), warrants further investigation. Resistance evolution is an important challenge to healthcare and food security alike. The pattern of accelerating resistance evolution we identified here for antifungals is certainly of enough significance to motivate studies to investigate how this pattern arises and should inform ways of reversing the trend. For antibiotics, the question of whether resistance evolution is accelerating needs to be urgently addressed. Even though the original data used in several references [4–7] could not be identified for antibiotics, it is possible that the trend observed in antifungals also applies to antibiotics. We have to assume that the estimates are based on expert knowledge. And almost certainly, data must exist and await being collated to investigate this pattern. The fact that antifungals show a pattern of accelerating resistance is of high importance itself because of their critical role in food production but should also serve as a sentinel for the study of antibiotic resistance evolution.

## Supporting information

S1 Fig The citation network behind the antibiotic introduction and resistance data. Sources that we could not acquire are indicated with *.
(TIF)

S2 Fig The citation network behind the antifungal introduction and resistance data. Sources that we could not acquire are indicated with *.
(TIF)

S1 Appendix Supplemental references for S1 Fig. (DOCX)

S2 Appendix Supplemental references for S2 Fig. (DOCX)

S1 Table Traced and complemented references for introduction and resistance emergence data for antifungals. (DOCX)
