# Supplementary figures and images for: Is antimicrobial resistance evolution accelerating?
Source: PLoS Pathog. 2020 Oct 22;16(10):e1008905. doi: 10.1371/journal.ppat.1008905 (PMC7580902; doi:10.1371/journal.ppat.1008905)

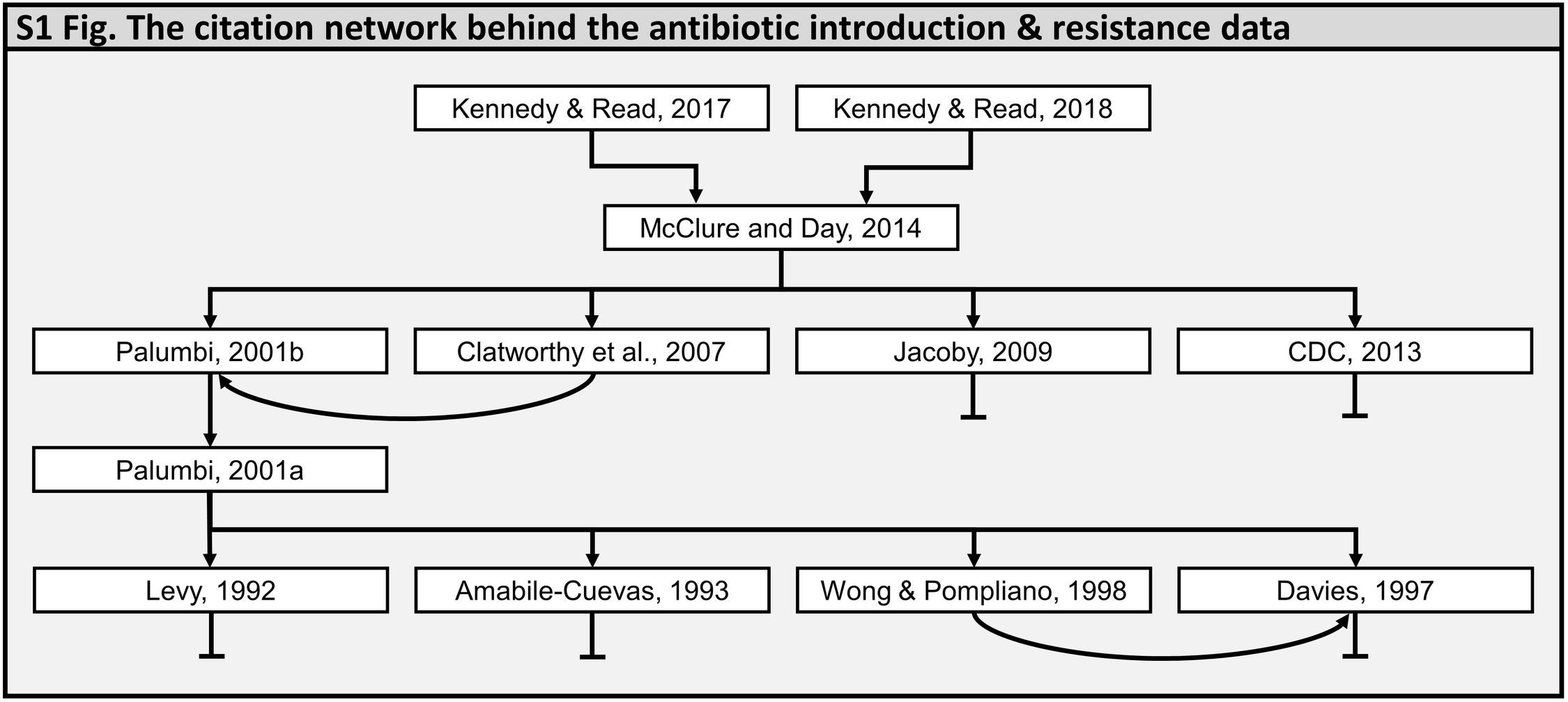

Supplement: S1 Fig — Sources that we could not acquire are indicated with *. (TIF) [file ppat.1008905.s001.tif]

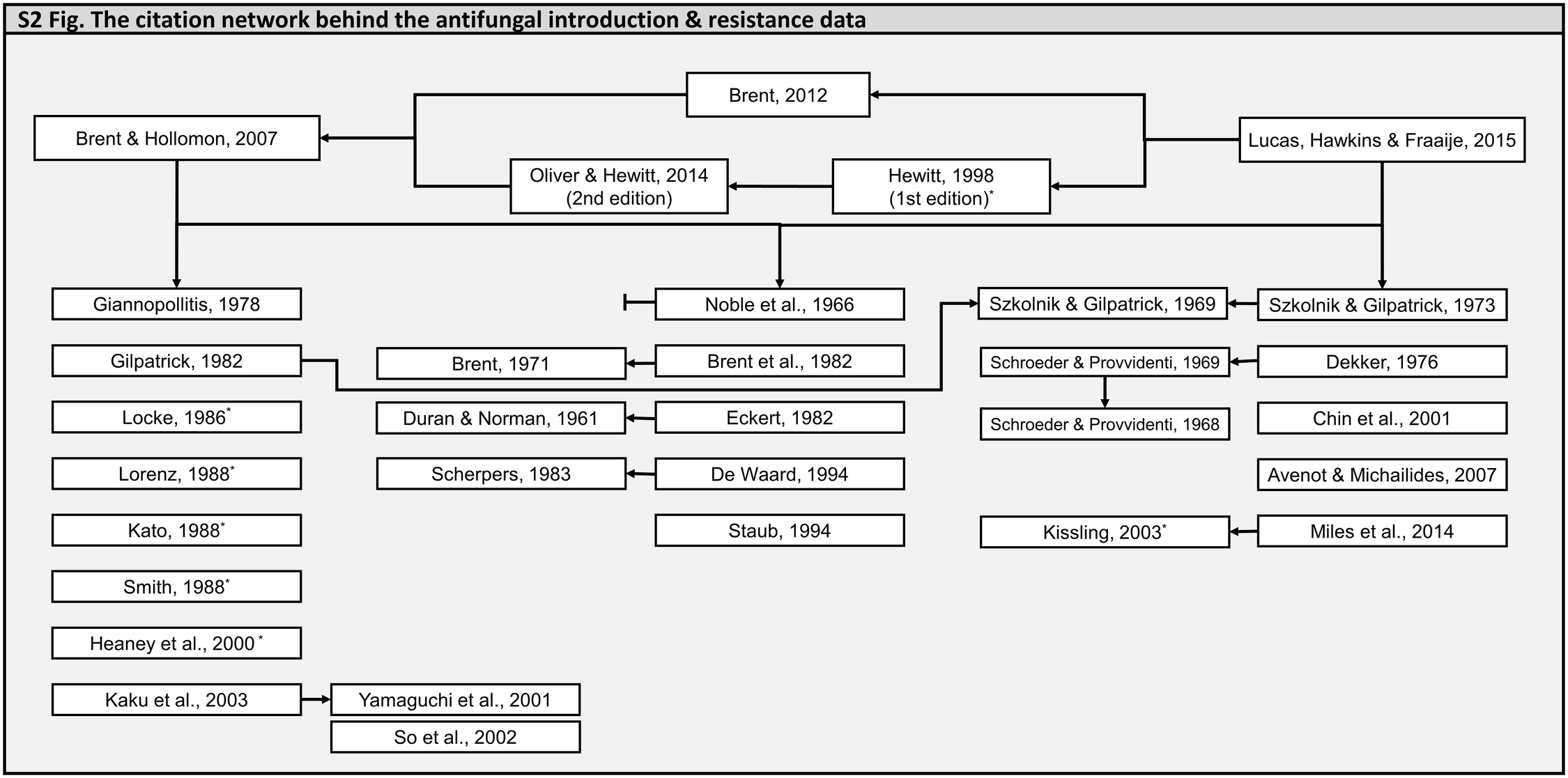

Supplement: S2 Fig — Sources that we could not acquire are indicated with *. (TIF) [file ppat.1008905.s002.tif]
