# Supplementary material for: Is antimicrobial resistance evolution accelerating?
Source: PLoS Pathog. 2020 Oct 22;16(10):e1008905. doi: 10.1371/journal.ppat.1008905 (PMC7580902; doi:10.1371/journal.ppat.1008905)
Supplement: S1 Appendix — (DOCX) [file ppat.1008905.s003.docx]

S1 Text. Supplemental References for S1 Figure.

Amábile-Cuevas CF. Origin, evolution and spread of antibiotic resistance genes. Austin Boca Raton, FL: R.G. Landes Co. ; Distributed worldwide by CRC Press; 1993.

Clatworthy AE, Pierson E, Hung DT. Targeting virulence: a new paradigm for antimicrobial therapy. Nat Chem Biol. 2007;3(9):541-8.

Davies JE. Origins, acquisition and dissemination of antibiotic resistance determinants. In: Chadwick D, Goode J, editors. Antibiotic resistance : origins, evolution, selection, and spread. Chichester ; New York: Wiley; 1997. p. 15-35.

Jacoby GA. History of Drug-Resistant Microbes. In: Mayers DL, editor. Antimicrobial Drug Resistance. Infectious Disease. Humana Press. 2009. p. 3-7.

Kennedy DA, Read AF. Why does drug resistance readily evolve but vaccine resistance does not? Proc Biol Sci. 2017;284(1851).

Kennedy DA, Read AF. Why the evolution of vaccine resistance is less of a concern than the evolution of drug resistance. Proc Natl Acad Sci U S A. 2018;115(51):12878-86.

Levy SB. The antibiotic paradox : how miracle drugs are destroying the miracle. New York: Plenum Press; 1992. p.47.

McClure NS, Day T. A theoretical examination of the relative importance of evolution management and drug development for managing resistance. Proc Biol Sci. 2014;281(1797).

Palumbi SR. Humans as the world's greatest evolutionary force. Science. 2001;293(5536):1786-90.

U. S. Department of Health and Human Services Centers for Disease Control and Prevention (2013). Antibiotic resistant threats in the United States. 2013. Retrieved from https://www.cdc.gov/drugresistance/threat-report-2013/pdf/ar-threats-2013-508.pdf

Wong KK, Pomploano DL. Peptidoglycan biosynthesis: Unexploited Antibacterial Targets within a Familiar Pathway. In: Rosen BP, Mobashery S, editors. Resolving the antibiotic paradox : progress in understanding drug resistance and development of new antibiotics. New York: Kluwer Academic/Plenum; 1998. p. 197-217.
