# Supplementary material for: Is antimicrobial resistance evolution accelerating?
Source: PLoS Pathog. 2020 Oct 22;16(10):e1008905. doi: 10.1371/journal.ppat.1008905 (PMC7580902; doi:10.1371/journal.ppat.1008905)
Supplement: S2 Appendix — (DOCX) [file ppat.1008905.s004.docx]

S2 Text. Supplemental References S2 Figure.

Avenot HF, Michailides TJ. Resistance to boscalid fungicide in Alternaria alternata Isolates from Pistachio in California. Plant Disease. 2007; 91(10), 1345–1350. http://dx.doi.org/10.1094/pdis-91-10-1345.

Brent KJ, Cole AM, Turner JAW, Woolner M. Resistance of cucumber powdery mildew to dimethirimol. In: Proceedings of the 6^th^ British Insecticide and Fungicide Conference. British Crop Protection Council, London. 1971. p. 274-282.

Brent KJ, Hollomon DW. Fungicide Resistance in Crop Pathogens: How can it be managed? 2nd rev. ed. Fungicide Resistance Action Committee; 2007

Brent KJ. Case study 4: powdery mildews of barley and cucumber In: Dekker J, Georgopoulos SG, editors. Fungicide resistance in crop protection. Wageningen, The Netherlands: Pudoc. 1982. p. 219–230.

Brent KJ. Historical perspectives of fungicide resistance. In: Thind TS, editor. Fungicide resistance in crop protection: Risk and management. Wallingford, UK: CABI; 2012. p. 3–18.

Chin KM, Chavaillaz D, Kaesbohrer M, Staub T, Felsenstein FG. Characterizing resistance risk of Erysiphe graminis f.sp tritici to strobilurins. Crop Protection. 2000; 20(2), 87–96. http://dx.doi.org/10.1016/s0261-2194(00)00059-4.

De Waard M. Resistance to fungicides which inhibit sterol 14a-demethylation, an historical perspective. In: Heaney S, Slawson D, Hollomon DW, Smith M, Russel PE, Parry DW, editors. Fungicide resistance. Farnham, Surrey, UK: British Crop Protection Council. 1994. p. 3-10.

Dekker J. Acquired-resistance to fungicides. Annual Review of Phytopathology. 1976; 14: 405–428. http://dx.doi.org/10.1146/annurev.py.14.090176.002201.

Duran R, Norman SM. Differential sensitivity to biphenyl among strains of Pénicillium digitatum Sacc. Plant Dis. Reptr. 1961; 45: 475-480.

Eckert JW. Case study 5: Penicillium decay of citrus fruits. In: Dekker J, Georgopoulos SG, editors. Fungicide resistance in crop protection. Wageningen, The Netherlands: Pudoc. 1982. p. 231–250.

Giannopolitis CN. Occurrence of strains of *Cercospora beticola* resistant to triphenyltin fungicides in Greece. Plant Disease Reporter. 1978; 62: 205-208.

Gilpatrick JD. Case study 2: Venturia of pome fruits and Monilinia of stone fruits. In: Dekker J, Georgopoulos SG, editors. Fungicide resistance in crop protection. Wageningen, The Netherlands: Pudoc. 1982. p. 195–206.

Heaney SP, Hall AA, Davis SA, Olaya G. Resistance to fungicides in the QoI-STAR cross-resistance group: current perspectives. Proceedings British Crop Protection Conference – Pests & Diseases. 2000, 755-762.

Hewitt HG. Fungicides in crop protection. Wallingford, Oxon, UK ; New York, NY: CAB International; 1998.

Kaku K, Takagaki M, Shimizu T, Nagayama K. Diagnosis of dehydratase inhibitors in melanin biosynthesis inhibitor (MBI-D) resistance by primer-introduced restriction enzyme analysis in scylatone dehydratase gene of *Magnaporthe grisea*. Pest Management Science; 2003; 59: 843-846.

Kato T. Resistance experiences in Japan. In: Charles JD, editor. Fungicide Resistance in North America. St Paul, Minnesota: American Phytopathological Society; 1988. p. 16-18.

Kissling E. EPA Registers Endura and Pristine Fungicides. Ludwigshafen, Germany: BASF SE. BASF News Release. 2003.

Locke T. Current incidence in the UK of fungicide resistance in pathogens of cereals. In: Proceedings British Crop Protection Conference, Pests & Diseases. 1986. p. 781-786.

Lorenz G. Dicarboximide fungicides: history of resistance development and monitoring methods. In: Charles JD, editor. Fungicide Resistance in North America. St Paul, Minnesota: American Phytopathological Society; 1988. p. 45-51.

Lucas JA, Hawkins NJ, Fraaije BA. The Evolution of Fungicide Resistance. In: Advances in Applied Microbiology. Elsevier; 2015. p. 29–92.

Miles TD, Miles LA, Fairchild KL, Wharton PS. Screening and characterization of resistance to succinate dehydrogenase inhibitors in Alternaria solani. Plant Pathology. 2014; 63(1), 155–164. http://dx.doi.org/10.1111/ppa.12077.

Noble M, Macqarvi QD, Hams AF, Leafe EL. Resistance to mercury of Pyrenophora avenae in scottish seed oats. Plant Pathology. 1966; 15(1): 23–28. http://dx.doi.org/10.1111/j.1365-3059.1966.tb00316.x.

Oliver RP, Hewitt HG. Fungicides in crop protection. 2nd edition. ed. Boston, MA: CABI; 2014.

Scherpers HTAM. Decreased sensitivity of Sphaerotheca fuliginea to fungicides which inhibit ergosterol biosynthesis. Neth. J. Pl. Path. 1983; 89, 185-187.

Schroeder WT, Provvidenti R. Resistance to benomyl in powdery mildew of cucurbits. Plant Dis. Reptr. 1969; 53:271-75O

Schroeder WT, Provvidenti R. Systemic Control of Powdery Mildew on Cucurbits with Fungicide 1991 applied as Soil Drenches and Seed Treatments. Plant Dis. Reptr. 1968; 52:630-632

Smith CM. History of benzimidazole use and resistance. In: Fungicide Resistance in North America. In: Charles JD, editor. Fungicide Resistance in North America. St Paul, Minnesota: American Phytopathological Society; 1988. p. 23-24.

So K, Fuji M, Iwabuchi H, Kanayama M, Yamaguchi J. Effects of various fungicides against less carpropamid-sensitive rice blast fungus isolated from the northwest area in Saga prefecture. Jpn J Phytopathol. 2002; 68:262. [in Japanese]

Staub T. Early experiences with phenylamide resistance and lessons for continued successful use. In: Heaney S, Slawson D, Hollomon DW, Smith M, Russel PE, Parry DW, editors. Fungicide resistance. Farnham, Surrey, UK: British Crop Protection Council. 1994. p. 131-138.

Szkolnik M, Gilpatrick J. Apparent resistance of Venturia inaequalis to dodine in New York apple orchards. Plant Dis. Reptr. 1969; 53:861-864.

Szkolnik M, Gilpatrick J. Tolerance of Venturia inaequalis to dodine in relation to history of dodine usage in apple orchards. Plant Disease Reporter. 1973; 57(10): 817–821.

Yamaguchi J, Kuchiki F, Hirayae K, So K. Decreased effect of carpropamid for rice blast control in the west north area of Saga prefecture in 2001. Jpn J Phytopathol. 2002; 68:261. [in Japanese]
