# Supplementary material for: Is antimicrobial resistance evolution accelerating?
Source: PLoS Pathog. 2020 Oct 22;16(10):e1008905. doi: 10.1371/journal.ppat.1008905 (PMC7580902; doi:10.1371/journal.ppat.1008905)
Supplement: S1 Table — (DOCX) [file ppat.1008905.s005.docx]

**S1 Table. Traced and complemented references for introduction and resistance emergence data for antifungals**

| antifungals | introduction | resistance | difference | reference_introduction | reference_resistance |
| --- | --- | --- | --- | --- | --- |
| Aromat Hydrocarbons | 1944 | 1961 | 17 | [1] | [2] |
| Organomercurials | 1924 | 1964 | 40 | No reference found | No reference found |
| Dodine | 1959 | 1969 | 10 | [3] | [3] |
| Benzimidazoles \| Benomyl | 1967 | 1968 | 1 | [4] | [5,6] |
| 2-Aminopyrimidines \| Dimethriol | 1969 | 1971 | 2 | [7] | [7,8] |
| Kasugamycin | 1965 | 1971 | 6 | [9] | [9,10] |
| Phosphorothiolates \| IBP | 1965 | 1976 | 11 | [11,12,13] | [12,14] |
| Triphenyltins | 1964 | 1977 | 13 | [15] | [15] |
| Phenylamides \| Metalaxyl | 1977 | 1980 | 3 | [16] | [16] |
| Dicarboximides | 1977 | 1980 | 3 | [17] | [17] |
| Sterol Demethylation Inhibitors \| Triforine | 1972 | 1982 | 10 | [18] | [19] |
| Oxathiins \| Carboxin | 1969 | 1985 | 16 | [20] | [21,22] |
| Quinone Outside Inhibitors | 1996 | 1998 | 2 | [23] | [23] |
| Melanin Biosynthesis Inhibitors \| Capropamid | 1998 | 2001 | 3 | [24,25] | [26,27,28] |
| Succinate Dehydrogensase Inhibitors \| Boscalid | 2003 | 2005 | 2 | [29] | [30] |
| Sterol Demethylation Inhibitors \| Biteranol | 1978 | 1987 | 9 | [31] | [31] |

**References**

1. Morton V, Staub T. A short History of Fungicides. Online, APS net Features. 2008. Doi: 10.1094/APSnetFeature-2008-0308.
2. Duran R, Norman SM. Differential sensitivity to biphenyl among strains of Pénicillium digitatum Sacc. Plant Dis. Reptr. 1961; 45: 475-480.
3. Szkolnik M, Gilpatrick J. Apparent resistance of Venturia inaequalis to dodine in New York apple orchards. Plant Dis. Reptr. 1969; 53:861-864.
4. Delp CJ, Klopping HL. Performance attributes of a new fungicide and mite ovicide candidate. Plant Dis. Reptr. 1968; 52:95-99.
5. Schroeder WT, Provvidenti R. Systemic Control of Powdery Mildew on Cucurbits with Fungicide 1991 applied as Soil Drenches and Seed Treatments. Plant Dis. Reptr. 1968; 52:630-632
6. Schroeder WT, Provvidenti R. Resistance to benomyl in powdery mildew of cucurbits. Plant Dis. Reptr. 1969; 53:271-75O
7. Brent KJ. Case study 4: powdery mildews of barley and cucumber In: Dekker J, Georgopoulos SG, editors. Fungicide resistance in crop protection. Wageningen, The Netherlands: Pudoc. 1982. p. 219–230.
8. Brent KJ, Cole AM, Turner JAW, Woolner M. Resistance of cucumber powdery mildew to dimethirimol. In: Proceedings of the 6^th^ British Insecticide and Fungicide Conference. British Crop Protection Council, London. 1971. p. 274-282.
9. Umezawa H, Okami Y, Hashimoto T, Suhara Y, Hamada M, Takeuchu T. A new antibiotic, Kasugamycin. J. Antibiot (Tokyo). 1965; 18:101-103.
10. Miura H, Ito H, Takahashi S. Occurrence of resistant strains of *Pyricularia oryzae* to kasugamycin as a cause of the diminished fungicide activity to rice blast. Ann Phytopathol Soc Jpn. 1975; 41:415–417. [in Japanese; abstract in English]
11. Bahadir M, Böger P, Buchenhauer H, Eto M, Khan MAQ, Pfister G, Sandmann G. Controlled release, biochemical effects of pesticides, inhibition of plant pathogenic fungi. Berlin ; New York: Springer-Verlag; 1990.
12. Katagiri M, Uesugi Y, Umehara Y. Development of resistance to organophosphorus fungicides in Pyricularia oryzae in the field. J Pestic Sci. 1980; 5:417–421.
13. Ishii H. Chapter 21: Rice Pathogens in Japan. In: Ishii H, Hollomon DW. Fungicide resistance in plant pathogens : principles and a guide to practical management. Tokyo: Springer; 2015.
14. Yaoita T, Go N, Aoyagi K, Sakurai H. Frequency distribution of sensitivity in rice blast fungus to an organophosphorus fungicide in Niigata Prefecture. Ann Phytopath Soc Jpn. 1978; 44: 401–402. [Abstr in Japanese]
15. Giannopolitis CN. Occurrence of strains of *Cercospora beticola* resistant to triphenyltin fungicides in Greece. Plant Disease Reporter. 1978; 62: 205-208.
16. Staub T. Early experiences with phenylamide resistance and lessons for continued successful use. In: Heaney S, Slawson D, Hollomon DW, Smith M, Russel PE, Parry DW, editors. Fungicide resistance. Farnham, Surrey, UK: British Crop Protection Council. 1994. p. 131-138.
17. Panayotakou M, Malathrakis NE. Resistance of *Botrytis cinereal* to dicarboximide fungicides in protected crops. Ann. Apl. Biol. 1983; 102, 293-299.
18. Scherpers HTAM. Changes during a three-year period in the sensitivity to ergosterol biosynthesis inhibitors of Sphaerotheca fuliginea in the Netherlands. Neth. J. Pl. Path. 1985; 91, 105-118.
19. Scherpers HTAM. Decreased sensitivity of Sphaerotheca fuliginea to fungicides which inhibit ergosterol biosynthesis. Neth. J. Pl. Path. 1983; 89, 185-187.
20. Stammler G, Wolf A, Glaettli A, Klappach K. Chapter 8: Respiration Inhibitors: Complex II. In: Ishii H, Hollomon DW. Fungicide resistance in plant pathogens : principles and a guide to practical management. Tokyo: Springer; 2015.
21. Leroux P. Caractéristiques des souches d’Ustilago nuda, agent du charbon nu de l’orge, résistantes à la carboxine. *Agronomie*. 1986; 6, 225-226. [in French]
22. Leroux P, Berthier G. Resistance to carboxin and fenfuram in Ustilago nuda (Jens.) Rostr., the causal agent of barley loose smut. Crop Protection. 1988; 7, 16-19
23. Chin KM, Chavaillaz D, Kaesbohrer M, Staub T, Felsenstein FG. Characterizing resistance risk of Erysiphe graminis f.sp tritici to strobilurins. Crop Protection. 2000; 20(2), 87–96. http://dx.doi.org/10.1016/s0261-2194(00)00059-4.
24. Kimura N, Fukuchi A. MBI-D resistance management of Pyricularia oryzae using an application program incorporating benomyl. J. Pestic. Sci. 2018; 43(1), 33-35.
25. Takagaki M. Chapter 11: Melanin Biosynthesis Inhibitors. In: Ishii H, Hollomon DW. Fungicide resistance in plant pathogens : principles and a guide to practical management. Tokyo: Springer; 2015.
26. Kaku K, Takagaki M, Shimizu T, Nagayama K. Diagnosis of dehydratase inhibitors in melanin biosynthesis inhibitor (MBI-D) resistance by primer-introduced restriction enzyme analysis in scylatone dehydratase gene of *Magnaporthe grisea*. Pest Management Science; 2003; 59: 843-846.
27. So K, Fuji M, Iwabuchi H, Kanayama M, Yamaguchi J. Effects of various fungicides against less carpropamid-sensitive rice blast fungus isolated from the northwest area in Saga prefecture. Jpn J Phytopathol. 2002; 68:262. [in Japanese]
28. Yamaguchi J, Kuchiki F, Hirayae K, So K. Decreased effect of carpropamid for rice blast control in the west north area of Saga prefecture in 2001. Jpn J Phytopathol. 2002; 68:261. [in Japanese]
29. U. S: Environmental Protection Agency; Office of Prevention, Pesticides and Toxic Substances. Fact Sheet for Boscalid. 2003. Retrieved from: https://www3.epa.gov/pesticides/chem_search/reg_actions/registration/fs_PC-128008_01-Jul-03.pdf
30. Avenot HF, Michailides TJ. Resistance to boscalid fungicide in Alternaria alternata Isolates from Pistachio in California. Plant Disease. 2007; 91(10), 1345–1350. http://dx.doi.org/10.1094/pdis-91-10-1345.
31. Braun PG. Development and decline of a population of Venturia inaequalis resistant to sterol-inhibiting fungicides. Norweg J Agric Sci. 1994; (Suppl 17): 173-184
